# Mesothelin expression correlates with elevated inhibitory immune activity in patients with colorectal cancer

**DOI:** 10.21203/rs.3.rs-3787873/v1

**Published:** 2023-12-28

**Authors:** Midhun malla, Sachin Kumar Deshkmukh, Sharon Wu, Timothy Samec, Dane Olevian, Reima Naili, El-Rayes Bassel, Joanne Xiu, Alex Farrell, Heinz-Josef Lenz, Emil Lou, Sanjay Goel, David Spetzler, Richard M. Goldberg, Lori Hazlehurst

**Affiliations:** University of Alabama; Caris Life Sciences; USC; West Virginia University

**Keywords:** Colorectal cancer, mesothelin, antigen-specific therapy, molecular oncology

## Abstract

The expression of the protein *Mesothelin (MSLN)* is highly variable in several malignancies including colorectal cancer (CRC) and high levels are associated with aggressive clinicopathological features and worse patient survival. CRC is both a common and deadly cancer; being the third most common in incidence and second most common cause of cancer related death. While systemic therapy remains the primary therapeutic option for most patients with stage IV (metastatic; m) CRC, their disease eventually becomes treatment refractory, and 85% succumb within 5 years. Microsatellite-stable (MSS) CRC tumors, which affect more than 90% of patients with mCRC, are generally refractory to immunotherapeutic interventions. In our current work, we characterize *MSLN* levels in CRC, specifically correlating expression with clinical outcomes in relevant CRC subtypes and explore how *MSLN* expression impacts the status of immune activation and suppression in the peritumoral microenvironment. High *MSLN* expression is highly prevalent in CMS1 and CMS4 CRC subtypes as well as in mCRC tissue and correlates with higher gene mutation rates across the patient cohorts. Further, *MSLN*-high patients exhibit increased M1/M2 macrophage infiltration, PD-L1 staining, immune-inhibitory gene expression, enrichment in inflammatory, TGF-β, IL6/JAK/STAT3, IL2/STAT5 signaling pathways and mutation in *KRAS* and *FBXW7*. Together, these results suggest *MSLN* protein is a potential target for antigen-specific therapy and supports investigation into its tumorigenic effects to identify possible therapeutic interventions for patients with high *MSLN* expressing MSS CRC.

## Introduction

In the United States, approximately 4% of people will develop colorectal cancer (CRC) at some point in their lifetime.^1,2^ In 2023, it is predicted that the incidence of CRC will be 153,000 new cases and over 52,500 deaths will occur in the US.^3^ Recently, the epidemiology of the disease has shifted with an increased incidence observed in individuals younger than 50 years. The average age of diagnosis was 72 in the early 2000s but has decreased to age 66 in the 2020s.^4^ As such, there are significant efforts to identify novel therapeutic targets and implement immunotherapies in CRC. Patients with CRC that has metastasized eventually become refractory to traditional systemic therapeutic approaches and succumb to the disease.^5^

Although there is a relatively high mortality rate associated with CRC, improvements in molecular characterization of tumors have permitted the development of novel treatment options for a subset of patients. These improvements have extended the median overall survival (OS) of patients with CRC from approximately 12 months to 25 to 30 months over the past 5 decades.^6,7^ Of note, molecular targets with therapeutic implications include adenomatous polyposis coli (*APC*), β-catenin, epithelial growth factor receptor (*EGFR*), V-Ki-ras2 Kirsten rat sarcoma viral oncogene homolog (*KRAS*), and *TP53*, among others.^8^ Targeting known drivers of cancer progression is critical to expanding the fraction of patients who may benefit from precision treatment of their CRC.^8^ Most recently, other genetic abnormalities, including the high frequency of *FBXW7* mutations, a finding that portends a poor prognosis, are under active investigation as additional potential molecular targets in CRC.^9^ However, a majority of patients with CRC have tumors that are microsatellite stable (MSS), a genotype which globally tends to be resistant to immunotherapies.^10^ With continued advancements in molecular characterization of MSS CRC tumors, it is hoped that methods of immunological targeting will be developed to improve upon current immunotherapy success levels in patients presenting with a MSS genotype.

A promising cellular therapeutic target under investigation is mesothelin (*MSLN*). *MSLN*, a protein with a suspected cell adhesion function in normal tissues, and is overexpressed in significant subsets of patients with numerous cancer types including mesothelioma, lung, pancreas, and CRC.^11–15^ Both preclinical and clinical studies examining *MSLN* expression in mesothelioma, pancreatic cancer, and lung adenocarcinoma show evidence that enhanced *MSLN* expression portends a reduction in survival in animals and humans.^13,14,16,17^ Specifically, in CRC, increased *MSLN* expression is correlated with the development of metastatic disease, a reduction in patient survival, and an upregulation of CRC cell proliferation.^11^ Numerous efforts to develop *MSLN* targeted therapies have been underway based on these preclinical data, specifically in mesothelioma, lung, and pancreatic cancers.^11–13,15–18^ However, work is still needed to exploit the therapeutic potential of high levels of *MSLN* expression in CRC.

This study seeks to correlate *MSLN* expression with known CRC prognostic signatures including tumor sidedness, metastatic sites, and the CRC consensus molecular subtype (CMS). Additionally, we seek to uncover associations of *MSLN* expression with specific CRC and immune-related molecular markers through examination of these relationships in all CRC patients and in the subset of MSS CRC patients. These interactions could identify new therapeutic options for MSS CRC patients with high levels of *MSLN* expression and lead to further investigations into therapeutics with clinical relevance to *MSLN* expression patterns.

## Methods

### Next Generation Sequencing (NGS)

NGS was performed on genomic DNA isolated from 14,892 formalin-fixed paraffin-embedded (FFPE) CRC tumor samples by a commercial CAP/CLIA lab (Caris Life Sciences, Phoenix, AZ) using the NextSeq or NovaSeq 6000 platforms (Illumina, Inc., San Diego, CA). The Caris platform uses a 592-gene panel and 700 gene panel at high depth and coverage as previously described.^19^ Tumor enrichment was performed using manual microdissection techniques. Genetic variants identified were interpreted by board-certified molecular geneticists and categorized as ‘pathogenic,’ ‘likely pathogenic,’ ‘variant of unknown significance,’ ‘likely benign,’ or ‘benign,’ according to the American College of Medical Genetics and Genomics (ACMG) standards. When assessing mutation frequencies of individual genes, ’pathogenic,’ and ‘likely pathogenic’ were counted as mutations.^20^ The copy number alteration (CNA) of each exon was determined by the average depth of the sample along with the sequencing depth of each exon and comparing to a pre-calibrated value.

### Tumor Mutation Burden (TMB)

TMB was measured by counting all mutations found per tumor that had not been previously denoted as germline alterations in dbSNP151, Genome Aggregation Database (gnomAD) databases or benign variants identified by Caris geneticists as previously described.^21^ A cutoff level of ≥ 10 mutations per MB was used to characterize tumors as TMB high based on evidence from the KEYNOTE-158 pembrolizumab trial which showed that patients with a TMB of ≥ 10 mt/MB across several tumor types had higher response rates than patients with a TMB of < 10 mt/MB.^22^

### Whole Transcriptome Sequencing (WTS)

A Qiagen RNA FFPE tissue extraction kit (Germantown, MD) was used for extraction, and the RNA quality and quantity were determined using the Agilent TapeStation (Santa Clara, CA) prior to WTS on the Illumina NovaSeq platform (Illumina, Inc., San Diego, CA) as previously described.^23^ For transcript counting, transcripts per million numbers were generated using the Salmon expression pipeline.^24^ RNA-deconvolution was performed via quanTIseq to assess immune cell infiltration within the tumor microenvironment (TME).^25^ Tumors were characterized as *MSLN*-high(H) and *MSLN*-low(L) based upon the top and bottom quartile of transcripts per million (TPM) expression, respectively. Gene set enrichment analysis (GSEA) was conducted in order to examine the enrichment or depletion of groups of genes associated with different biological pathways. This analysis was performed using Broad Institute software.^26^

### Immunohistochemistry

Immunohistochemistry (IHC) of PD-L1 (SP142 clone), MLH1 (M1 clone), MSH2 (G2191129 clone), MSH6 (44 clone), and PMS2 (EPR3947 clone) was completed on FFPE tissue slides. Slides were stained as per the manufacturer’s instructions (Ventana Medical Systems, Inc. Tucson, AZ), and were optimized and validated per CLIA/CAP and ISO requirements. Staining was scored for intensity (0 = no staining; 1 + = weak staining; 2 + = moderate staining; 3 + = strong staining) and staining percentage (0–100%). The complete absence of protein expression of any of the 4 proteins tested (0 + in 100% of cells) was considered deficient MMR. A board-certified pathologist evaluated all IHC results independently.

### MSI/MMR Status

Multiple test platforms were used to determine the MSI or MMR status of the tumors profiled, including fragment analysis (FA, Promega, Madison, WI), IHC (see IHC method) and NGS (7,000 target microsatellite loci were examined and compared to the reference genome hg19 from the University of California). The three platforms generated highly concordant results, as previously reported. In the rare cases of discordant results, the MSI or MMR status of the tumor was determined in the order of IHC, FA and NGS.^27^

### MPAS

MAPK activation score (MPAS) was calculated based on the TPM values of RNA expression of *CCND1, DUSP4, DUSP6, EPHA2, EPHA4, ETV4, ETV5, PHLDA1, SPRY2*, and *SPRY4* using a previously reported algorithm as a transcriptomic indicator of MAPK pathway activation.^28^

### CODEai^™^

Insurance claims data were used to calculate real-world overall survival (rwOS) via ‘first of treatment’ to ‘last contact’ patient records. Kaplan-Meier estimates were calculated for molecularly defined patient cohorts.

### Statistical analysis

Statistical significance was determined using Chi-square and Mann-Whitney U tests, with p values adjusted for multiple comparisons (q ≤ 0.05). rwOS significance was determined with p ≤ 0.05.

## Results

### MSLN Cohort Demographics and Expression Distribution in Primary Tumors, Metastatic Site, Tumor Side, and CMS Subtypes

The study population comprised 7,446 patients, of which 6,847 patients were assigned to the “MSS cohort” according to MSI status. Both the entire patient cohort and MSS cohort were dichotomized as *MSLN* Low or *MSLN* High. Shown in Table 1A, the *MSLN* low group included 3,723 patients with 54.9% being male and 45.1% female. The *MSLN* high group included 3,723 patients with 52.9% being male and 47.1% female. The median age was 63 years and 62 years respectively. In Table 1B, the *MSLN* low cohort included 3,377 patients, with a median age of 62 years with 56.5% being male and 43.5% female. The *MSLN* high group was comprised of 3,470 patients, 53.7% of whom were male and 46.3% female. The median age was 62 years for both *MSLN* expression levels in the MSS cohort.

**Figure 1:**
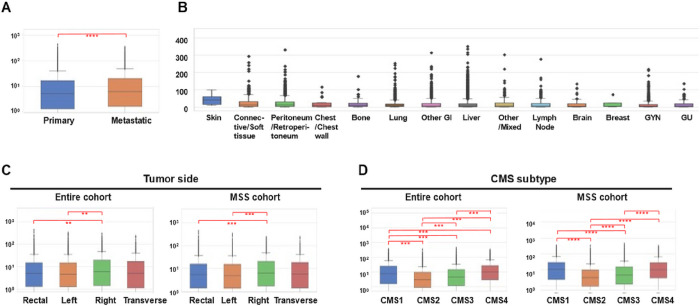
*MSLN* expression in CRC is enhanced in metastatic tissue, right-sided CRC, and CMS1 and CMS4 molecular subtypes. *MSLN* median TPM expression was analyzed in primary and metastatic CRC (A), various sites of CRC metastasis (B), primary tumor sidedness across patient cohorts (C) and CMS subtypes across patient cohorts (D). * q < 0.05; ** q < 0.01; *** q < 0.001, **** q < 0.0001.

*MSLN* expression patterns were compared across primary and metastatic sites, metastatic location, CRC side of origin, and CMS subtypes (Fig. 1). Figure 1A and 1B show *MSLN* expression in the primary tumor versus metastatic sites in all patients. Overall, tumor samples from metastatic sites expressed *MSLN* at significantly higher levels compared to the primary tumor site (q ≤ 0.01) (Fig. 1A). Samples from metastases to the skin (39.4 TPM), connective/soft tissue (11.9 TPM), and the peritoneum/retroperitoneum (11.7 TPM) exhibit the highest MSLN expression levels amongst metastatic sites (Fig. 1B). Shown in Fig. 1C, *MSLN* expression was highest in right sided CRC tumors in both the entire cohort (6.1 TPM), and MSS (6.2 TPM), cohorts (q ≤ 0.01). Additionally, *MSLN* expression was high in transverse tumor locations in both patient cohorts. Left sided tumors exhibited the lowest median *MSLN* expression levels at 4.8 TPM. Similar expression patterns between the entire and MSS cohorts were observed when comparing CMS subtypes (Fig. 1D). Both cohorts exhibited the highest *MSLN* expression in CMS1 (8.3 and 10.4 TPM respectively, q ≤ 0.001) and CMS4 (10.1 and 10.0 TPM respectively, q ≤ 0.001).

### MSLN Expression is Associated with Increased Mutations in Several Cancer Associated Genes

**Figure 2:**
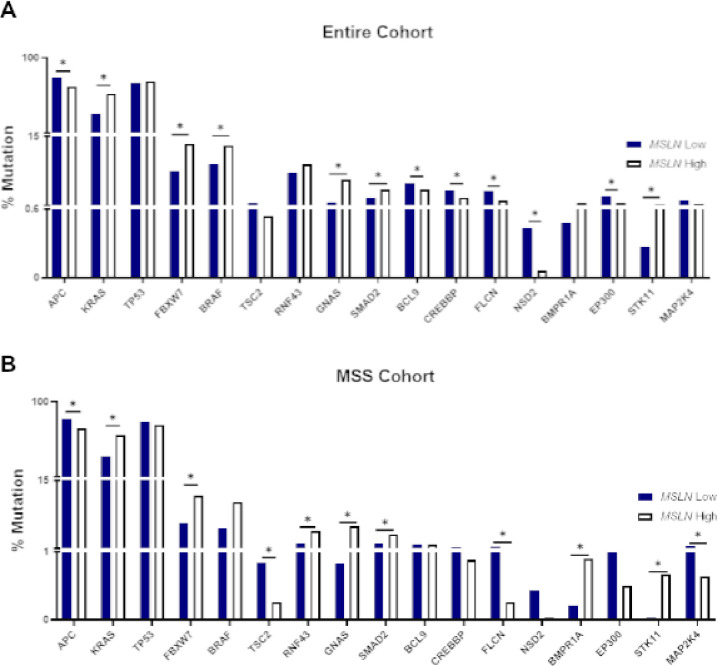
*MSLN* expression yields high rates of oncogenic driver mutations. Mutation analysis was performed in CRC samples expressing low and high *MSLN* across entire cohort (A) and MSS cohort (B). * p < 0.05.

Numerous oncogenic driver mutation rates and their relation to *MSLN* gene expression were explored. Within the entire and MSS patient cohorts, comparisons were made between *MSLN* high expressing tissues and *MSLN* low expressing tissues (Fig. 2). High and low expression quartiles were determined as described in the methods above. The entire cohort of patients (Fig. 2A) displayed significant differences for numerous gene mutations between the *MSLN* high and low expression cohorts. APC (p = 1.7e-20), *BCL9* (p = 0.0006), *CREBBP* (p = 8.5e-5), *FLCN* (p = 1.39e-7), *NSD2* (p = 0.002), and *EP300* (p = 1.42e-6) all had significantly higher mutation rates in *MSLN* low expressing tumor samples compared to *MSLN* high expressing tumor samples (Fig. 2A). Conversely, *KRAS* (p = 2.67e-90), *FBXW7* (p = 1.29e-14), *BRAF* (p = 9.32e-8), *GNAS* (p = 2.54e-29), and *SMAD2* (p = 3.93e-5) mutated tumors were significantly more common in *MSLN* high expressing tumor samples versus *MSLN* low expressing tumor samples (Fig. 2A). These mutation patterns were quite similar in the MSS cohort. *APC* (p = 8.35e-26), *TSC2* (p = 0.001), *FLCN* (p = 3.77e-7), and *MAP2K4* (p = 0.002) were significantly more likely to be mutated in *MSLN* low tumor samples whereas *KRAS* (p = 2.58e-89), *FBXW7* (p = 8.38e-16), *RNF43* (p = 4.06e-10), *GNAS* (p = 4.36e-29), *SMAD2* (p = 4.7e-6), *BMPR1A* (p = 1e-4), and *STK11* (p = 8.61e-6) were significantly more likely to be mutated in *MSLN* high tumor samples (Fig. 2B). Of note, APC, *KRAS* and TP53, regardless of patient cohort, exhibited high mutation rates of at least 30% in each *MSLN* expression quartile. Full mutation data is presented in supplementary table 1.

### Mesothelin High Tumors Exhibit Greater Expression of PD-L1 and Higher T-cell Inflamed Score, Immune Cell Infiltration, and Expression of Immunosuppressive Genes

**Figure 3:**
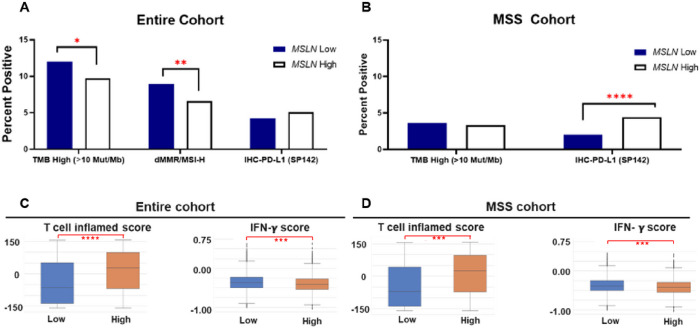
MSS tumors with high *MSLN* expression yield high PD-L1 staining and T-cell inflammation. Tumor mutation burden (TMB), dMMR/MSI-high status and PD-L1 percent positivity (A), T-cell inflamed score (B) and IFN-y score (C) analysis was performed in MSLN low and high CRC. * q < 0.05; ** q < 0.01; *** q < 0.001.

Figure 3 examines the prevalence of immune markers across *MSLN* high and low tissue expression split into the two cohorts as mentioned previously. Across the entire cohort (Fig. 3A), *MSLN* low expression had a significant association with higher tumor mutation burden (TMB) and DNA mismatch repair (dMMR)/microsatellite instability-high (MSI-H) positivity in comparison to *MSLN* high expression. However, *MSLN* high expression exhibited an association with high PD-L1 expression via IHC (IHC-PD-L1) compared to MSLN low expression. In the MSS cohort, *MSLN* high tumor samples yielded significantly higher IHC-PD-L1 positivity compared to *MSLN* low expression (Fig. 3B). Figure 3C and 3D show similar results regarding T-cell inflammation quantification and IFN-γ scores. Across both cohorts it was found that *MSLN* high expression significantly correlated with both markers of T-cell inflammation and low IFN-γ scores.

**Figure 4:**
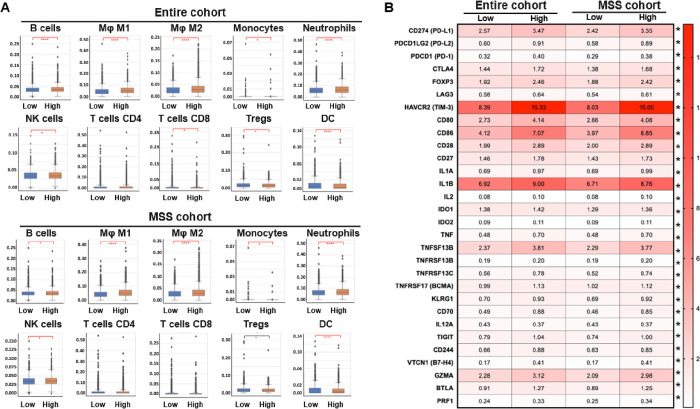
Significant immune cell fractions in *MSLN* high CRC are found in B cells macrophages, neutrophils, NK cells, and T-regs regardless of patient cohort. (A) Immune cell fractions were estimated by RNA-seq using Quantiseq. (B) Immune-related gene expression were analyzed in MSLN low and high CRC. * q < 0.05; ** q < 0.01; *** q < 0.001.

Specific immune cell infiltration into the TME (Fig. 4A) and immune gene expression (Fig. 4B) was examined within each cohort in addition to the immune marker results presented in Fig. 3. Several immune cell types including B cells, M1 and M2 macrophages, neutrophils, natural killer (NK) cells, and regulatory T-cells (Tregs) were more prevalent in *MSLN* high expressing tumors across both the entire cohort and MSS cohort. Specifically, M1 and M2 macrophages were most significant within these groups. Only dendritic cells (DC) were more prevalent in *MSLN* low tumors. Full immune cell fraction data values are presented in supplementary table 2. Immune marker gene expression levels, shown in Fig. 4B, were all higher in *MSLN* high tumors across both cohorts except for *IL12A*. Particularly, *HAVCR2* (TIM-3), *CD80*, *CD86*, and *IL1B* expression were of highest magnitude change between MSLN high and low expressing tumors. In the entire cohort of patients, comparing *MSLN* high and *MSLN* low groups, *HAVCR2* (15.33 TPM vs. 8.39 TPM, q ≤ 0.05), *CD80* (4.14 TPM vs. 2.73 TPM, q ≤ 0.05), *CD86* (7.07 TPM vs. 4.12 TPM, q ≤ 0.05), and *IL1B* (9.00 TPM vs. 8.76 TPM, q ≤ 0.05) were all significantly higher in the *MSLN* high group. A relationship in *HAVCR2* expression was seen in the MSS cohort between *MSLN* high and low tissue (15.00 TPM vs 8.03 TPM, q ≤ 0.05). In the MSS cohort, *MSLN* high tissue again yielded increased gene expression in *HAVCR2* (15.00 TPM vs. 8.03 TPM, q ≤ 0.05), *CD80* (4.08 TPM vs. 2.66 TPM, q ≤ 0.05), *CD86* (8.76 TPM vs. 3.97 TPM, q ≤ 0.05), and *IL1B* (8.76 TPM vs. 6.71 TPM, q ≤ 0.05).

### Gene Set Enrichment Analysis of MLSN High Tumors

**Figure 5:**
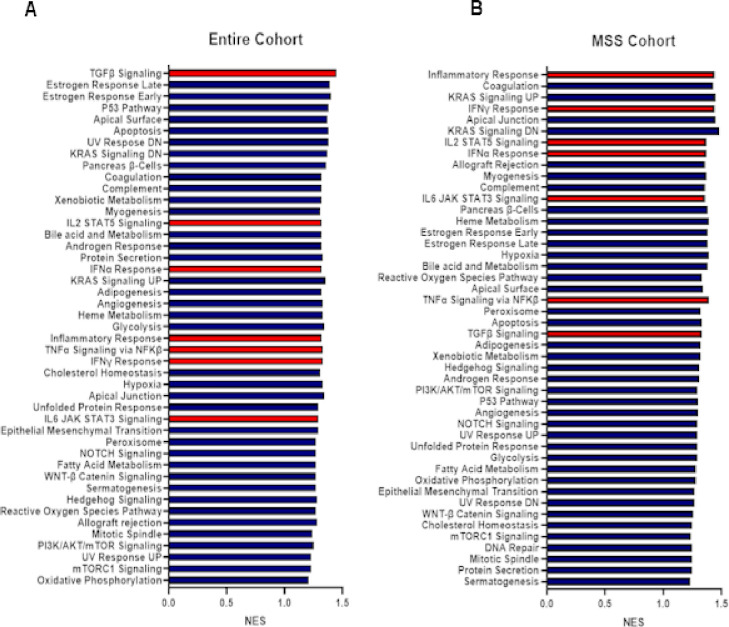
MSS CRC with high *MSLN* exhibits significant gene enrichment for inflammation and immune response. Gene set enrichment analysis was performed in *MSLN* low and high CRC entire cohort (A) and MSS cohort (B). Where FDR ≤ 0.25 considered significant.

Association of immune cell recruitment, tumor microenvironment, and corresponding gene sets related to immune response can support the possibility of a gene target being a marker for tumor susceptibility to antigen-specific immunotherapy. Complementing the immune marker and microenvironment analyses, a Gene Set Enrichment Analysis (GSEA) was performed to evaluate the differences in *MSLN*-associated gene expression between patients with *MSLN* low and *MSLN* high tumors within each cohort. For all gene sets shown (Fig. 5A, B), a positive normalized enrichment score (NES) was observed, indicating higher gene enrichment for patients with *MSLN* high tumors. Each cohort of patients exhibited significantly high NES for gene sets related to immune response related to *MSLN* high expression, shown using red bars. Specifically, TNFα signaling via NFKβ (NES = 1.33, False Discovery Rate (FDR) = 0.09), IL2 STAT5 signaling (NES = 1.32, FDR = 0.08), IFN-γ response (NES = 1.33, FDR = 0.09), and IL6 JAK STAT3 signaling (NES = 1.29, FDR = 0.12) were three such pathway enrichments related to immune response that were significantly enriched in patients with *MSLN* high tumors in contract with those who had *MSLN* low tumors. In the MSS cohort, it must first be noted that one of the most highly enriched pathways was that of the inflammatory response (NES = 1.44, FDR = 0.01). Additionally, immune-related pathways including IFN-γ Response (NES = 1.44, FDR = 0.02), IL2 STAT 5 Signaling (NES = 1.37, FDR = 0.06), IFNα Response (NES = 1.37, FDR = 0.07), IL6 JAK STAT3 Signaling (NES = 1.36, FDR = 0.07), and TNFα Signaling via NFKβ (NES = 1.39, FDR = 0.09) were all highly significant in the MSS cohort. These pathways were more significant based on FDR in the MSS cohort than in the entire cohort, possibly suggesting a higher role of immune modulation in patients with MSS tumors.

### Patient Outcomes in Association with MSLN Expression

**Figure 6:**
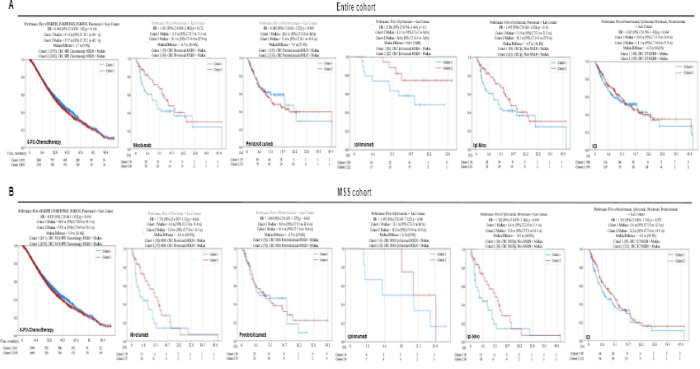
Patient survival outcomes improve for MSLNhigh MSS CRC when treated with nivolumab and pembrolizumab. Kaplan-Meier curves depicting survival from time of tissue collection or first treatment to last contact for entire cohort (A) and MSS cohort (B) of CRC patients with *MSLN* low (blue; cohort 1) compared to *MSLN* high (red; cohort 2).

Survival outcomes relative to *MSLN* expression levels were analyzed using Caris CODEai^™^. Patient outcomes in each cohort of patients (MSS cohort and entire cohort) have been compared across various treatment modalities based on chemotherapy (5-fluorouracil(FU) based) or immune checkpoint inhibitory (ICI) therapy (nivolumab (nivo), ipilimumab (ipi), and pembrolizumab (pembro). Both the entire cohort (Fig. 6A) and MSS cohort of patients (Fig. 6B) displayed similar patterns in that *MSLN* high expression had an associated lower survival than patients whose tumors were *MSLN* low in the 5-FU based chemotherapy treated group. In the entire cohort, nivolumab (18.1 months vs 11.4 months, HR = 1.433, 95% CI 0.854–2.403, p = 0.172), ipilimumab (Inf vs 21.1 months, HR = 2.596, 95% CI 0.798–8.444, p = 0.1, and the combination Ipi-Nivo (18.1 months vs 11.4 months, HR = 1.447, 95% CI 0.863–2.426, p = 0.16) were the exceptions in that patients with *MSLN* high tumors survived a longer median number of days compared to those with *MSLN* low tumors (Fig. 6A). In the MSS cohort, patients with *MSLN* high tumors treated with immunotherapy exhibited better survival. Patients with tumors exhibiting *MSLN* high expression survived a median of 13.0 months versus 4.6 months in those with *MSLN* low tumors when treated with nivolumab (HR = 1.731, 95% CI 0.957–3.13, p = 0.068 Fig. 6B). Patients treated with pembrolizumab whose tumors exhibited *MSLN* high expression survived a median 10.6 months while patients with *MSLN* low tumors survived a median of 8.0 months (HR = 1.044, 95% CI 0.651–1.675, p = 0.86, Fig. 6B). The Ipi-Nivo combination therapy resulted in a median survival for patients with *MSLN* high tumors of 13.0 months versus those with *MSLN* low tumors who had a median survival time of 4.6 months (HR = 1.762, 95% CI 0.975–3.186, p = 0.059, Fig. 6B). Finally, ICI treatment in *MSLN* high tumor patients yielded a median survival time of 12.2 months, while *MSLN* low tumor patients survived a median of 8.0 months (HR = 1.221, 95% CI 0.855–1.743, p = 0.272, Fig. 6B).

### Survival Outcomes in patients treated with immune checkpoint inhibitors, analyzed based on MSLN expression quartiles

**Figure 7:**
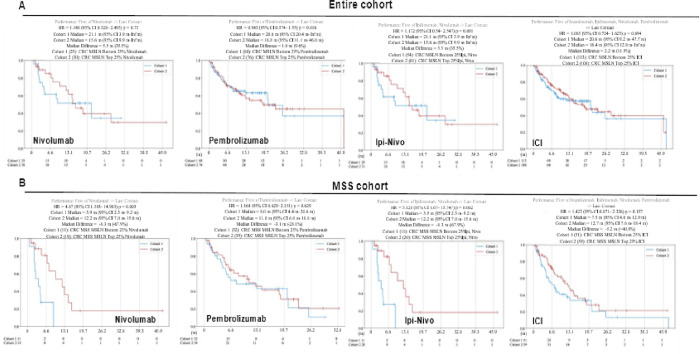
Patient survival outcomes improve in MSLN high tumors with MSS status across all immunotherapeutic treatment strategies. Kaplan-Meier curves generated via CODEai^™^ in CRC patients within the entire cohort (A) and MSS cohort (B) by the lowest 25% *MSLN*expression quartile (blue/cohort 1) and highest 25% *MSLN* expression quartile (red/cohort 2).

Survival outcomes were again analyzed via Caris CODEai^™^ using the highest and lowest 25% *MSLN* expression quartiles to further evaluate the correlation of *MSLN* expression with treatment efficacy. Each cohort, the entire cohort (Fig. 7A) and the MSS cohort (Fig. 7B), were categorized by treatment intervention, all being immunotherapeutic in nature: nivolumab, pembrolizumab, Ipi-Nivo combination, and all ICI combined. For the entire cohort (Fig. 7A), patients with tumors in the lowest 25% *MSLN* expression (blue) had a longer median survival time than patients with tumors exhibiting the highest 25% *MSLN* expression (red) in nivolumab (21.1 months vs 15.6 months; HR 1.18, 95% CI 0.528–2.495, p = 0.73), pembrolizumab (20.6 months vs 18.8 months; HR 0.94, 95% CI 0.574–1.55, p = 0.816), Ipi-Nivo combination (21.1 months vs 15.6 months; HR 1.172, 95% CI 0.54–2.547), and the combination of each ICI (20.6 months vs 18.4 months; HR 1.08, 95% CI 0.724–1.625, p = 0.694) treatment groups. In the MSS patient cohort (Fig. 7B), the opposite effect of ICI appeared to be present. Across nivolumab, pembrolizumab, Ipi-Nivo, and all ICI, the patients with the highest 25% *MSLN* expression in their tumors (red) survived longer than the patients with tumors exhibiting the lowest 25% *MSLN* expression (blue). Median survival times for nivolumab were 3.9 months and 12.2 months for the bottom and top 25% *MSLN* expression groupings, respectively (HR = 4.87, 95% CI 1.583–14.983, p = 0.003). In the pembrolizumab treated group, median survival times were 8.0 months and 11.1 months for the bottom and top 25% *MSLN* expression cohorts respectively (HR = 1.164, 95% CI = 0.629–2.151, p = 0.629). The combined Ipi-Nivo treatment yielded survival times of 3.9 months and 12.2 months for the bottom 25% of *MSLN* expressing cohort and the top 25% of *MSLN* expressing cohort, respectively (HR = 5.128, 95% CI 0.67–15.747, p = 0.002). In the combined ICI curve, patients with tumors exhibiting the lowest 25% *MSLN* expression survived a median 7.5 months those whose tumors had the highest 25% *MSLN* expression survived a median 12.7 months (HR = 1.423, 95% CI 0.871–2.326, p = 0.157). Both the cohorts with high MSLN expression who were treated with nivolumab had significantly longer survival times at p = 0.003.

## Discussion

Colorectal cancer is the third most common cause of cancer and second highest cause of cancer related mortality in the United States.^29^ There is significant heterogeneity among patients with CRC in terms of genomic abnormalities that drive tumor progression and these differences correlate with prognosis. By establishing connections between novel biomarkers and prognostic signatures of CRC including tumor sidedness, metastatic sites, CMS category, and immune cell recruitment, we may be able to preferentially select treatments for patients with enhanced expression patterns of these biomarkers, possibly leading to an improvement in the survival outcomes. Mesothelin has been shown to play a role in promoting cancer cell survival, proliferation, and invasion of cancer cell lines enabling cancer cells to thrive in an inflammatory milieu.^17,30,31^ Our work begins to uncover the relevance of *MSLN* overexpression in CRC patients as well as the implications of MSLN expression levels on pivotal CRC prognostic information including CRC tumor sidedness, CMS subtype, genetic mutation landscape, and immune response modulation. We have shown that increased *MSLN* expression is positively correlated with right-sided CRC, CMS1, and CMS4 molecular subtypes, possibly playing a role in the poor prognosis associated with right-sided CRC and CMS1/4 molecular subtypes.^32,33^ Currently, it is understood that CMS1 is characterized by increased immune-related gene expression, including activation of immune evasion that is commonly observed in MSI CRC, with high rates of right-sided tumors and poor survival rates with disease relapse.^34^ CMS2 is typically categorized by upregulation of WNT and MYC, leading to carcinogenesis, and enhanced epithelial cell differentiation.^34^ CMS3 often is seen with mixed MSI status but high rates of *KRAS* mutation and CMS4 is associated with high rates of epithelial-to-mesenchymal transition (EMT) gene expression in addition to activation of *TGF*-β and inflammatory pathways, contributing to the poorest overall survival among CMS subtypes.^34^ Regarding tumor sidedness, previous work has shown that right-sided tumors are typically associated with a poorer 5-year survival (55% vs 59%) and an increased risk of CRC recurrence versus left-sided tumors.^35,36^

As stated, our results show an increased association of CMS1, CMS4, and right-sided CRC with high *MSLN* expression. Due to this relationship, it can be posited that this increase in *MSLN* gene expression does also play a role in the associated features of these molecular subtypes and tumor location. The immune and inflammatory enhancements seen with CMS1 and CMS4 CRC are mirrored in the increased immune cell recruitment, immune-related gene expression, and enhancement of immune and inflammatory pathways shown with high *MSLN* expression (Figs. 4, 5). Further, CMS4 has specifically been linked to EMT enhancements creating desirable conditions for rapid tumor growth and metastatic activity.^37^ It is plausible that with increased MSLN expression, specifically in CMS4, there may be a link between *MSLN* expression and EMT. Bringing focused attention to the MSS cohort of patients and the relationship between MSLN and EMT activation, we have shown that high *MSLN* expression in this subset of patients exhibit increased TGF-β signaling (Fig. 5B), *KRAS* signaling (Fig. 5B), MAPK activation (Supp. Figure 1), and significant enrichment of the EMT gene set (Fig. 5B).^38,39^ Each of these factors, in addition to associated increased CA125/*MUC16* expression (Supp. Figure 2), an *MSLN* binding domain, which has also been shown to play a role in tumorigenesis and *STAT3* phosphorylation^40^, support the consideration of *MSLN* as a prospective biomarker in CRC.

Directly related to the potential clinical relevance of *MSLN* as a biomarker is the difference in survival time for patients with tumors exhibiting high *MSLN* expression versus low *MSLN* expression across multiple therapeutic regimens. Illustrated in Fig. 6, CODEai^™^ analysis revealed some significant differences across these patient cohorts, specifically in patients with tumors classified as MSS. MSS tumors that had high *MSLN* expression had a worse associated median survival time when treated with 5-FU-chemotherapy and ipilimumab alone (Fig. 6B). Notably, there was a shift in this pattern when patients with *MSLN* high tumors that were also MSS exhibited a higher median survival time than the *MSLN* low cohort when treated with nivolumab, pembrolizumab, Ipi-Nivo combination, or the ICI combination. By determining *MSLN* expression oncologists may choose to recommend treatment with ICI and that strategy may enhance survival outcomes. The patients with highest and lowest quartiles of *MSLN* expression were also investigated using CODEai^™^ analysis (Fig. 7). The lowest 25% of *MSLN* expressing tumors in the entire cohort showed longer survival times than the tumors with the highest 25% of *MSLN* expression when treated with different forms of ICI (Fig. 7A). What is most noteworthy, is that when we analyzed the cohort of patients with tumors classified as MSS, once again, the survival patterns shifted, with the *MSLN* high cohorts surviving longer than the *MSLN* low cohort across all ICI treatment groups (consisting of nivolumab, pembrolizumab, Ipi-Nivo, and combination ICI treatment) (Fig. 7B). It is this combination of high *MSLN* expression and MSS status that yields an improved survival with ICI treatment that implicates *MSLN* as a possible biomarker in MSS CRCs. Moreover, it may be suggested that this improved survival outcome with ICI in patients with *MSLN* high MSS CRC is due to the immunosuppressive TME induced by increased *MSLN* expression.

Our study has demonstrated a strong association between *MSLN* expression and a differentially modulated immune microenvironment, highlighted by an increase in M1/M2 macrophage recruitment, but a lowered IFN-γ score and general immunosuppressive effect, though pathway activation is shown in GSEA analysis. It should also be emphasized that high *MSLN* expression, especially in MSS patients, was associated with significantly increased IHC-PD-L1 staining (Fig. 3B) and enhanced gene expression of PD-1, CTLA4, FOXP3, LAG3, and TIM3 (Fig. 4B). Further, total gene set enrichment analysis revealed significant activation of gene sets associated with TGF-β signaling, IL6/JAK2/STAT pathway signaling, IL2/STAT5 pathway signaling, and PIK3/AKT/mTOR signaling (Fig. 5B). These findings are supported by other studies that have described the effects of *MSLN*-related immunosuppression and tumor immune escape in other cancers via upregulation of immunosuppressive genes and cytokine production.^41,42^ Immune cell response, relative ‘heat’ of the tumor microenvironment, immune/inflammatory pathway activations, and genetic mutation profiles may predict the likelihood of benefit from immunotherapy in relation to *MSLN* expression, specifically in patients with MSS tumors. Regarding gene mutation patterns, recent studies have determined that increased mutation in *KRAS* and *FBXW7* have been implicated as immunosuppressive biomarkers that play a role in poor immunotherapeutic responses in CRC.^43,44^ Additionally, high co-expression of *HAVCR2* and *MSLN* have been shown to be prevalent in several cancers and may also be vulnerabole to the use of CAR-T therapies. Pre-clinical studies have shown the utility of CAR-T therapy in an *MSLN* high ovarian cancer model.^45^ Taking this deeper into the *HAVCR2-MSLN* relationship, RNA-interference therapies have also been used in combination with CAR-T approaches to first knock down *HAVCR2*/TIM3 expression and enhance the cytotoxic functioning and proliferation capacity of therapeutic CAR-T cells in numerous *in vitro* cancer models.^46^ TIM-3 has been shown to be a candidate for gene knockdown to enhance cell-based immunotherapy previously.^47^ Taking these studies together, in combination with our conclusions of *MSLN* high implications on patient response to immunotherapies, may yield a prospective combination therapy approach. In *MSLN* high expressing tumors that are MSS, we have reported significantly increased mutation rates in *KRAS* and *FBXW7* as opposed to our findings in *MSLN* low expressing patient cohorts, suggesting that *MSLN* status may also be relevant to the modulation of the TME and immune response in CRC. Taking this one step further, the top quartile of *MSLN* expressing tumor cohorts exhibited significantly longer survival times, again supporting *MSLN* as a potential biomarker. We have also demonstrated (Figs. 6, 7) that a subset of patients, particularly the MSS subset, show an *MSLN* expression-dependent response to ICI therapies, highlighting the prospect of *MSLN* as a clinically relevant biomarker for therapeutic selection. However, it is recommended that additional pre-clinical testing using genetic strategies to modulate levels of *MSLN* to be completed to determine if any causative relationship exists between MSLN expression and modulation of the TME towards immune suppression in MSS CRC. Improved survival in *MSLN* high CRCs post-ICI could be attributed to increased PD-L1 protein, *CD274* (PD-L1), *PDCD1* (PD-1), and *CTLA4* gene expression, and enrichment of inflammatory response pathways in these tumors. However, the small size of the ICI therapy cohorts for survival analysis is one of the limitations of this study, which will require further validation in larger studies. In addition, data on tumor stage and grade were available in a limited number of patients included in our analysis and, therefore, we were also unable to determine the relationship of these prognostic variables with treatment outcomes. Future prospective analysis will be required to answer such important questions. Further studies should be performed to fully uncover the implication of *MSLN* expression in CRC patients, specifically MSS patients, to improve the efficacy and availability of therapeutic options to a patient population that currently sees limited benefit beyond progression on the current standard of care treatment options.

## Figures and Tables

**Table 1:** Demographic patient data by *mesothelin (MSLN)* expression level in the entire cohort of patients (A) and MSS cohort of patients (B).

A		
Entire cohort	MSLN Low	MSLN High
**Count (N)**	3723	3723
**Median age (range)**	63 [18 - >89]	62 [18 - >89]
**Male**	2044 (54.9%)	1969 (52.9%)
**Female**	1679 (44.7%)	1754 (47.0%)
B		
MSS	MSLN Low	MSLN High
**Count (N)**	3377	3470
**Median age (range)**	62 [18 - >89]	62 [18 - >89]
**Male**	1908 (56.5%)	1862 (53.7%)
**Female**	1469 (43.5%)	1608 (46.3%)

## Data Availability

The datasets generated during and/or analyzed during the current study are available from the corresponding author on reasonable request. The deidentified sequencing data are owned by Caris Life Sciences. Qualified researchers can apply for access to these summarized data by contacting Joanne Xiu, PhD and signing a data usage agreement.
